# Increased expression of long non-coding RNA CCEPR is associated with poor prognosis and promotes tumorigenesis in urothelial bladder carcinoma

**DOI:** 10.18632/oncotarget.17872

**Published:** 2017-05-15

**Authors:** Yonghao Zhan, Yifan Li, Bao Guan, Xiaoying Chen, Zhicong Chen, Anbang He, Shiming He, Yanqing Gong, Ding Peng, Yuchen Liu, Zhiming Cai, Xuesong Li, Liqun Zhou

**Affiliations:** ^1^ Department of Urology, Peking University First Hospital, The Institute of Urology, Peking University, National Urological Cancer Centre, Beijing, 100034, China; ^2^ Department of Urology, State Engineering Laboratory of Medical Key Technologies Application of Synthetic Biology, Key Laboratory of Medical Reprogramming Technology, Shenzhen Second People's Hospital, The First Affiliated Hospital of Shenzhen University, Shenzhen, 518035, China

**Keywords:** bladder cancer, biomarker, lncRNA CCEPR, PCNA, tumorigenesis

## Abstract

Recent emerging evidences have showed that long non-coding RNAs play important regulatory roles in diverse biological processes of tumor development and progression. CCEPR (cervical carcinoma expressed PCNA regulatory lncRNA) is a novel identified lncRNA that acts as a potential biomarker and involves in development and progression of cervical carcinoma. Nevertheless, we know nothing about the clinical significance and molecular mechanism of CCEPR in bladder cancer. In this study, we found that CCEPR was significantly up-regulated in bladder cancer. Furthermore, up-regulated CCEPR expression was positively correlated with advanced TNM stage and higher histological grade. Moreover, further experiments demonstrated that CCEPR promotes cell proliferation and suppresses cell apoptosis in bladder cancer. Mechanistically, we found CCEPR upregulates the expression of PCNA in mRNA and protein level to promote cancer growth. In conclusions, these findings demonstrated that CCEPR plays an important regulatory role in bladder cancer and may serve as a potential diagnostic biomarker and therapeutic target.

## INTRODUCTION

Urothelial carcinoma of the bladder is one of most common malignancy all over the world and the most common urologic tumors in China [1, 2]. The incidence and mortality of bladder cancer have been significantly increased in the past decade [3–6]. Despite improvements in current clinical treatment such as surgery, adjuvant chemoradiotherapies, and immunological therapy, the prognosis of patients has not been significantly improved [7–11]. Patient's prognosis is closely related to the stage of the disease, but there are no efficient methods for diagnosis at early stage [12, 13]. Therefore, more sensitive and specific markers for diagnosis at early stage and more efficient and safer treatments are urgently needed [14].

LncRNAs are a class of noncoding RNAs which are greater than 200 nucleotides in length and limited coding potential [15–17]. The rapid development of human genomics has highlighted the important role of non-coding RNAs in diverse biological processes of cancer [18–20]. Recent emerging evidences have shown that lncRNAs play key roles in development and progression of bladder cancer, such as UCA-1, PVT-1, MALAT1, SPRY4-IT1, PANDAR and etc [21–30]. CCEPR (cervical carcinoma expressed PCNA regulatory lncRNA, GenBank number AK055418) is a novel identified lncRNA with 2504 nucleotides in length and localized at the chromosome 10 [20]. Recently, CCEPR originally was identified as a powerful tumor biomarker for cervical cancer [31]. However, its biological function in bladder cancer development is still completely unknown.

Although CCERP has been shown to serve as an oncogene, the underlying molecular mechanism of CCERP in tumorigenesis remains to be clarified. The function of lncRNAs usually relies on the proteins that they interact with. For example, the lncRNA HOTTIP has been reported to directly interact with the WDR5 protein and target WDR5/MLL complexes [32]. The lncRNA PANDAR has also been reported to interact with the NF-YA to repress gene regulation [33]. And the lncRNA HNF1A-AS1 has also been reported to mediate the binding of DNMT1 to E-cadherin [26, 34]. Yang and colleagues have provided evidence that CCEPR associates with PCNA (proliferating cell nuclear antigen) mRNA, consequently increases the expression level of PCNA [31]. We speculated that CCERP may play a similar role in bladder cancer cell.

In this study, we found that the expression of CCEPR is significantly increased in bladder cancer tissues and cell lines, moreover, CCEPR promotes proliferation and suppresses apoptosis of bladder cancer cells *in vitro*. Our results suggested that CCEPR plays an key role and may serve as a promising diagnostic and therapeutic target for bladder cancer.

## RESULTS

### The expression of CCEPR is increased in bladder cancer

**Figure 1:**
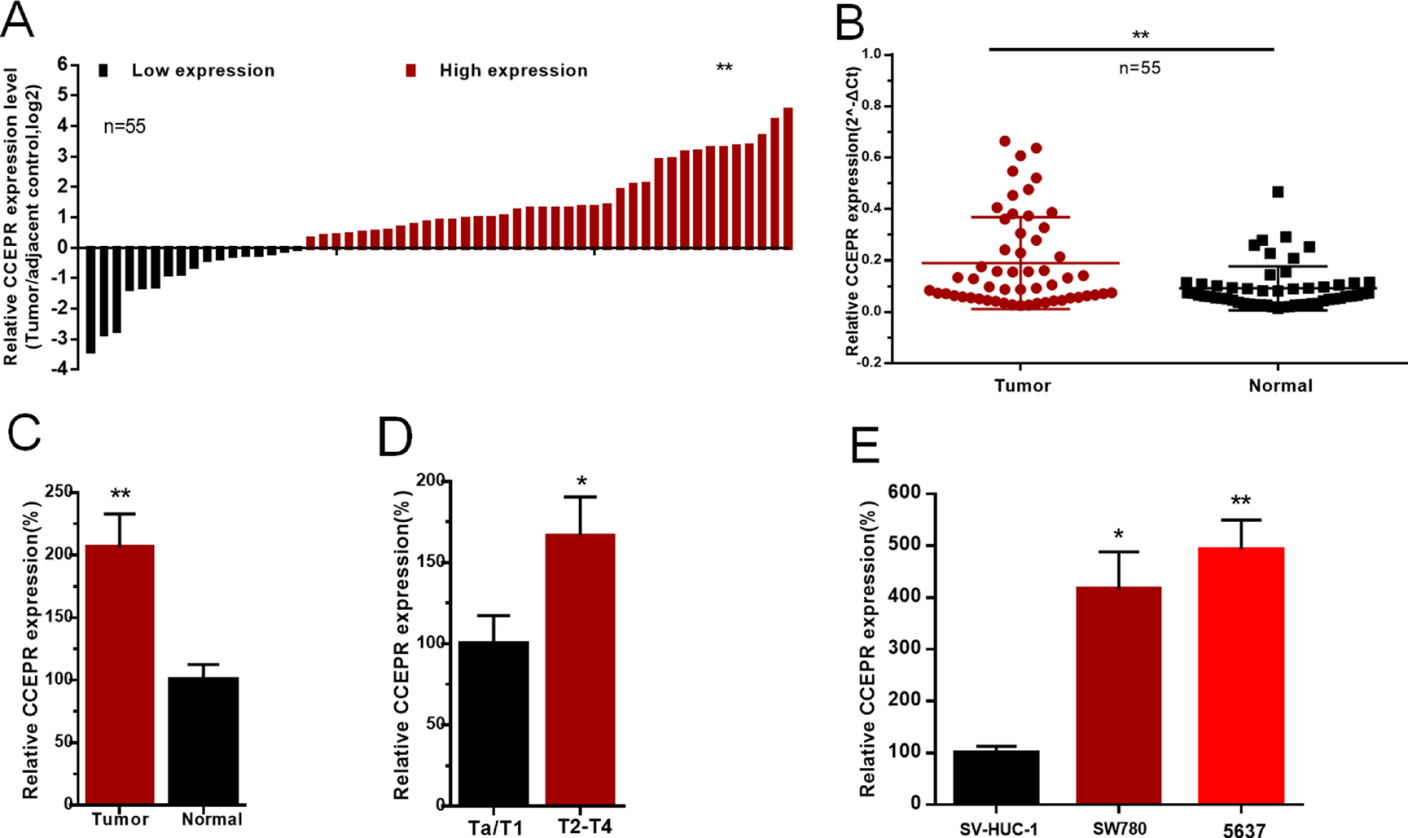
The relative expression levels of CCEPR in bladder cancer (**A**) The heights of the columns in the chart represent the log2-transformed fold changes (bladder cancer tissue/normal bladder tissue) in CCEPR expression in 55 patients with bladder cancer. (**B** and **C**) CCEPR is up-regulated in bladder cancer tissues compared with corresponding non-tumor tissues. (**D**) CCEPR was up-regulated in patients with advanced TNM stage. (**E**) CCEPR was up-regulated in bladder cancer cell lines. Data are shown as mean ± SD. *p < 0.05; **p < 0.01.

The expression of CCEPR in bladder cancer tissues and cell lines was detected by qRT-PCR. CCEPR mRNA levels were signifcantly up-regulated in bladder cancer tissues compared to corresponding non-tumor tissues (Figure 1A, 1B, 1C). Furthermore, elevated expression of CCEPR was positively associated with advanced TNM stage(Figure 1D). CCEPR mRNA levels was up-regulated in bladder cancer cell lines (Figure 1E). Statistical results and clinicopathological features of 55 patients are shown in Table 1 and Supplementary Table 1, respectively.

**Table 1:** Correlation between CCEPR expression and clinicopathological features of UCB patients

Parameters Total	Group	Total	CCEPR expression		*P* value
			High	Low	
Gender	Male	40 (73%)	27 (49%)	13 (24%)	0.677
	Female	15 (27%)	11 (20%)	4 (7%)	
Age (years)	< 60	20 (36%)	15 (27%)	5 (9%)	0.473
	≥ 60	35 (64%)	23 (42%)	12 (22%)	
Tumor size (cm)	< 3 cm	21 (38%)	15 (27%)	6 (11%)	0.768
	≥ 3 cm	34 (62%)	23 (42%)	11 (20%)	
Multiplicity	Single	32 (58%)	25 (45%)	7 (13%)	0.087
	Multiple	23 (42%)	13 (24%)	10 (18%)	
Histological grade	L	23 (42%)	12 (22%)	11 (20%)	0.021*
	H	32 (58%)	26 (47%)	6 (11%)	
Tumor stage T	Ta, T1	14 (26%)	6 (11%)	8 (15%)	0.014*
	T2–T4	41 (74%)	32 (58%)	9 (16%)	

*P < 0.05 was considered significant (Chi-square test between 2 groups).

### Corresponding specific siRNA/pcDNA3.1 down/up-regulated expression level of CCEPR

**Figure 2:**
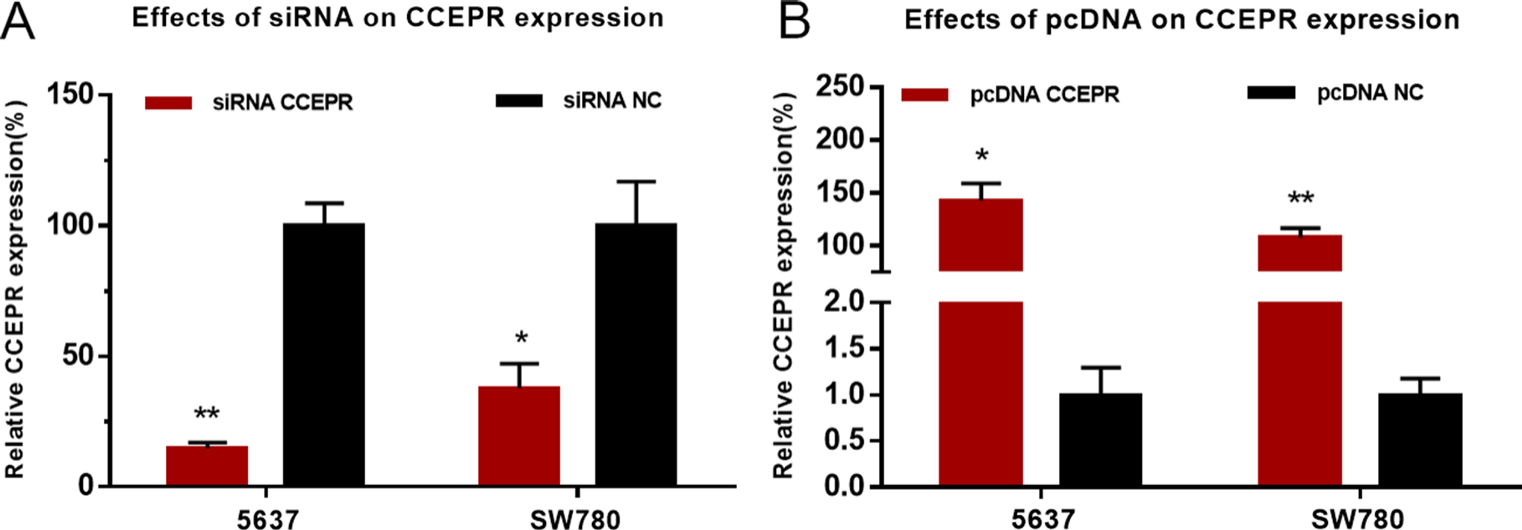
Effects of corresponding siRNA or pcDNA on CCEPR expression level (**A**) The CCEPR specific siRNA significantly down-regulated the expression level of CCEPR in 5637 and SW780 cells. (**B**) The CCEPR specific pcDNA3.1 significantly up-regulated the expression level of CCEPR in 5637 and SW780 cells. Data are indicated as mean ± SD. *p < 0.05; **p < 0.01.

5637 and SW780 cells were cultured and then transfected with CCEPR specific siRNA or expression vector. The results of qRT-PCR showed that CCEPR mRNA levels in 5637 and SW780 cells was significantly decreased by siRNA-CCEPR (Figure 2A) and increased by pcDNA3.1-CCEPR (Figure 2B).

### CCEPR promotes the proliferation of bladder cancer cells

**Figure 3:**
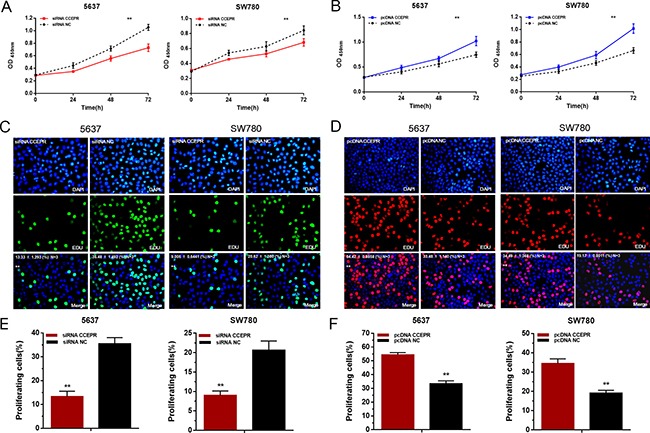
The effect of CCEPR on bladder cancer cell proliferation (**A**, **C** and **E**) Silencing CCEPR inhibited cell proliferation in 5637 and SW780 cells. (**B**, **D** and **F**) Overexpressing CCEPR promoted cell proliferation in 5637 and SW780 cells. Data are shown as mean ± SD. *p < 0.05; **p < 0.01.

The cell proliferative capacity were evaluated by Cell Counting Kit-8 assay and Ethynyl-2-deoxyuridine incorporation assay. Silencing CCEPR inhibited cell proliferation in 5637 and SW780 cells (Figure 3A, 3C, 3E). Overexpressing CCEPR promoted cell proliferation in 5637 and SW780 cells (Figure 3B, 3D, 3F).

### CCEPR promotes cell cycle and increases the expression of PCNA

**Figure 4:**
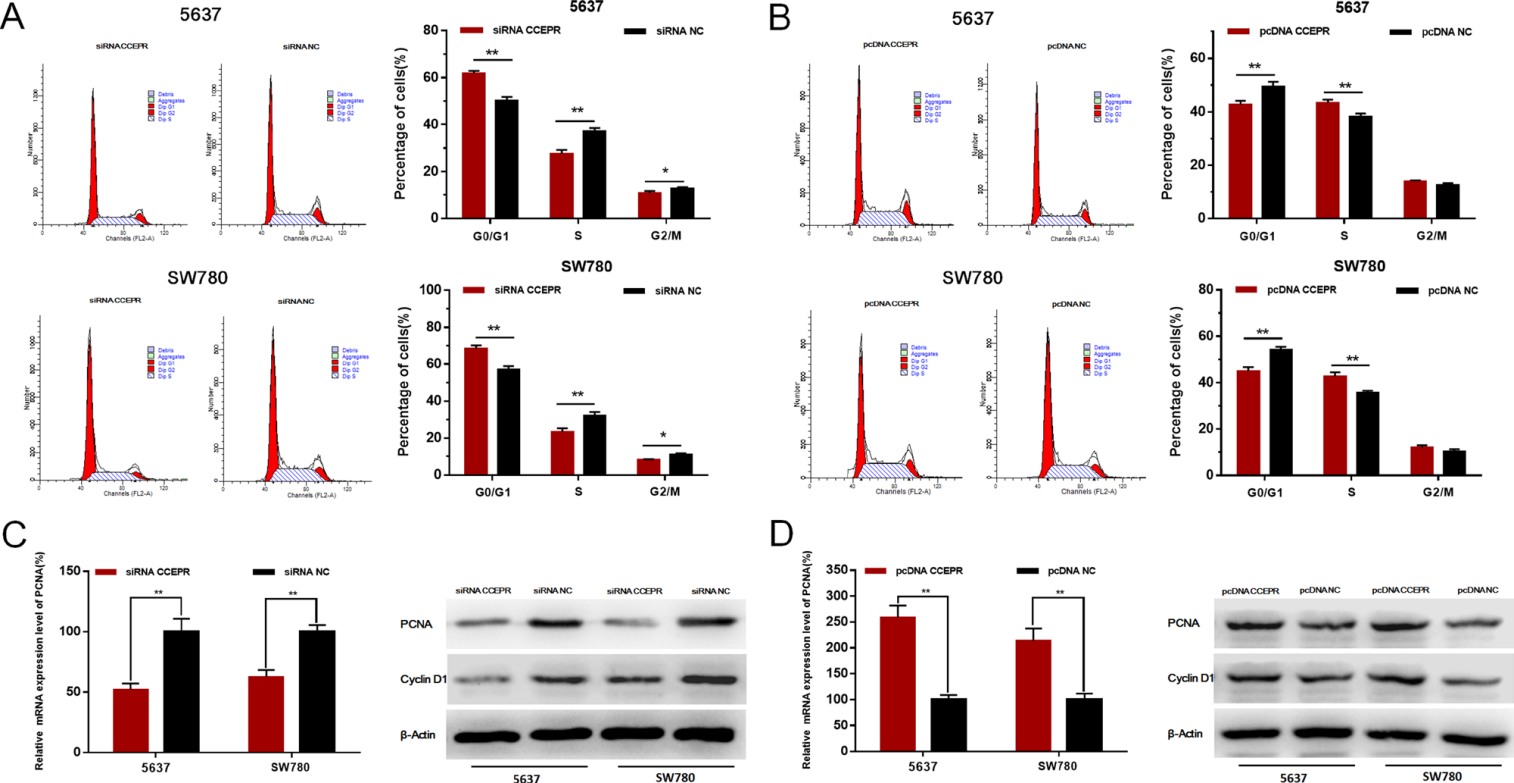
The effect of CCEPR on bladder cancer cell cycle and the expression of PCNA (**A**) Silencing CCEPR inhibited cell cycle in 5637 and SW780 cells. (**B**) Overexpressing CCEPR promoted cell cycle in 5637 and SW780 cells. (**C**) Down-regulation of CCEPR decreased the mRNA and protein level of PCNA. (**D**) Up-regulation of CCEPR increased the mRNA and protein level of PCNA. Data are shown as mean ± SD. *p < 0.05; **p < 0.01.

The cell cycle was evaluated by Flow cytometry. Silencing CCEPR inhibited cell cycle in 5637 and SW780 cells (Figure 4A). Overexpressing CCEPR promoted cell cycle in 5637 and SW780 cells (Figure 4B). To investigate the underlying mechanisms of CCEPR-mediated biological processes, we performed bio-information analysis, qRT-PCR and western blotting. As shown in Figure 4C and 4D, down-regulation of CCEPR significantly decreased PCNA mRNA and protein levels and up-regulation of CCEPR significantly increased PCNA mRNA and protein levels in 5637 and SW780 cells.

### CCEPR suppresses the apoptosis of bladder cancer cells

**Figure 5:**
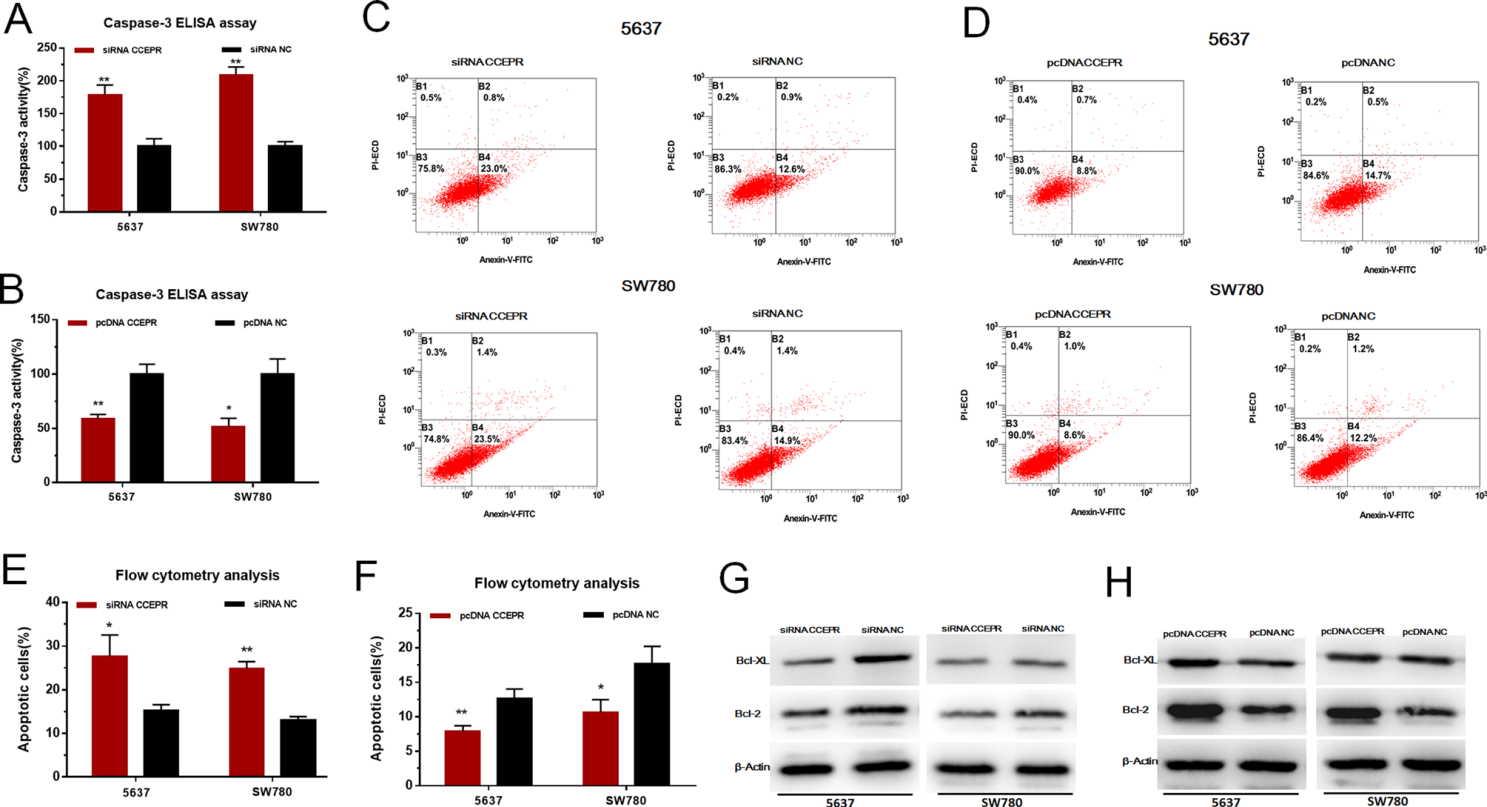
The effect of CCEPR on bladder cancer cell apoptosis (**A**, **C** and **E**) Silencing CCEPR induced cell apoptosis in 5637 and SW780 cells. (**B**, **D** and **F**) Overexpressing CCEPR suppressed cell apoptosis in 5637 and SW780 cells. (**G**) Down-regulation of CCEPR decreased the anti-apoptosis associated markers (Bcl-2/Bcl-XL). (**H**) Up-regulation of CCEPR increased the anti-apoptosis associated markers (Bcl-2/Bcl-XL). Data are shown as mean ± SD. *p < 0.05; **p < 0.01.

We further evaluated the apoptosis of bladder cancer cells by ELISA assay and Flow cytometry. Silencing CCEPR induced cell apoptosis in 5637 and SW780 cells (Figure 5A, 5C, 5E). Overexpressing CCEPR suppressed cell apoptosis in 5637 and SW780 cells (Figure 5B, 5D, 5F). As shown in Figure 5G, 5H, Down-/Up-regulation of CCEPR decreased /increasedthe anti-apoptosis associated markers (Bcl-2/Bcl-XL).

### CCEPR does not regulate cell migration and invasion in bladder cancer

**Figure 6:**
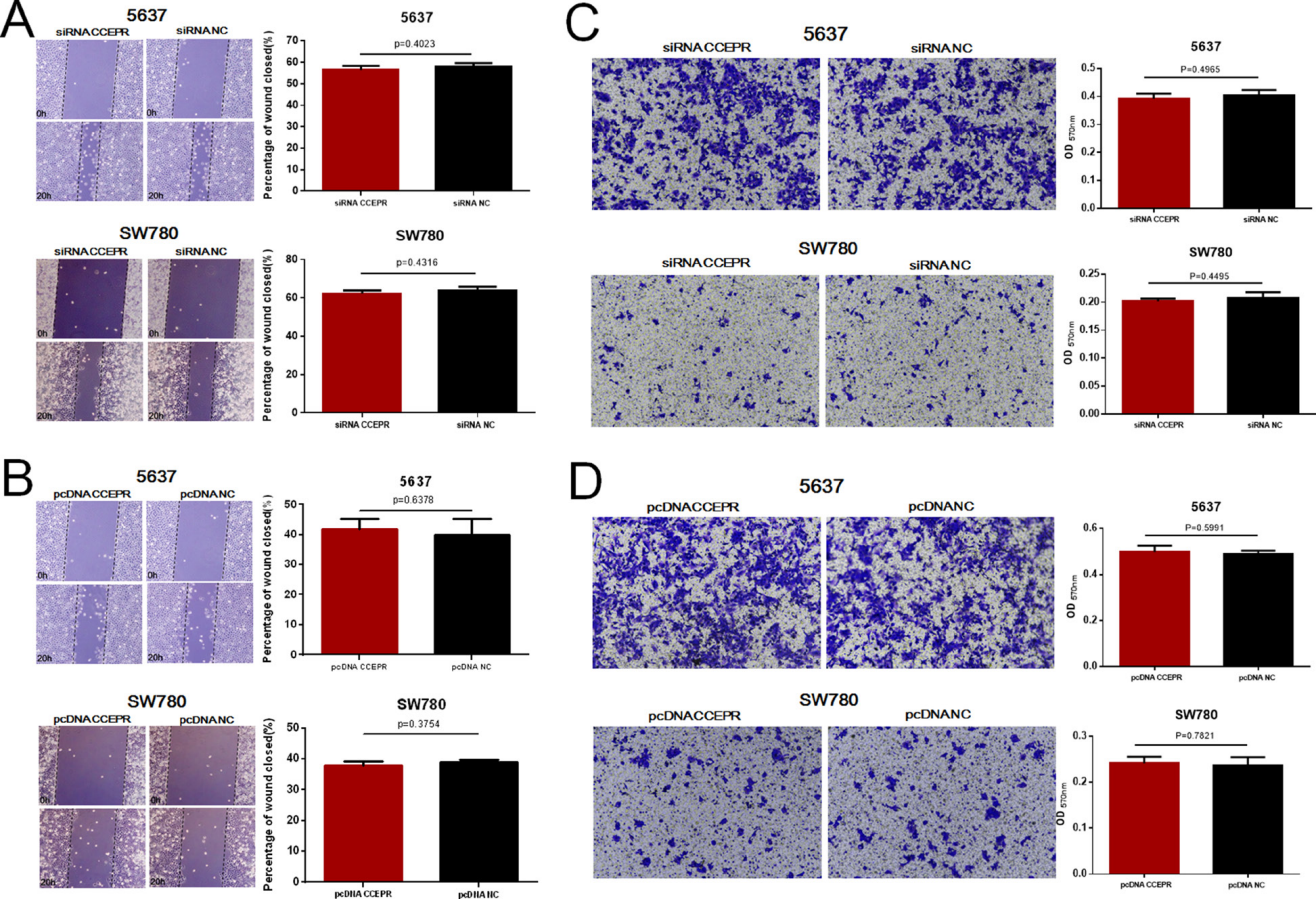
The effect of CCEPR on bladder cancer cell metastasis (**A** and **B**) There was no significant difference in the migratory ability of bladder cancer cells transfected with corresponding specific siRNA/pcDNA3.1. (**C** and **D**) There was no significant difference in the invasive ability of bladder cancer cells transfected with corresponding specific siRNA/pcDNA3.1. Data are shown as mean ± SD. *p < 0.05; **p < 0.01.

We further evaluated whether CCEPR regulates the migration and invasion of bladder cancer cells. Regrettably, there was no significant difference in the migratory and invasive abilities of cells transfected with CCEPR specific siRNA (Figure 6A, 6C) or pcDNA3.1 (Figure 6B, 6D). The results indicated that CCEPR does not regulate cell migration and invasion in bladder cancer.

## DISCUSSION

Urothelial carcinoma of the bladder is the most common genitourinary malignancies and a major cause of morbidity and mortality in China [35, 36]. Because the molecular mechanisms of tumorigenesis in bladder cancer are still unknown, the therapeutic outcomes for patients with bladder cancer remain unsatisfactory [37, 38]. Therefore, to explore detailed molecular mechanisms of bladder cancer development and progression is essential in improving the clinical strategies and outcomes of bladder cancer [39, 40].

The lncRNAs which are longer than 200 nucleotides are a class of noncoding RNAs [41]. Recently, an increasing number of evidences indicated that lncRNAs play important roles in cancer development and progression [42–44]. CCEPR was previously reported to interact with PCNA and regulate cervical carcinoma occurrence and progression [31]. However, the biological functions and underlying mechanisms of CCEPR in bladder cancer are still unknown.

In this study, we found the expression level of CCEPR is significantly increased in bladder cancer and CCEPR could promote proliferation and suppresse apoptosis in bladder cancer cells. Mechanistically, the function of lncRNAs usually relies on the proteins that they interact with. We found CCEPR upregulates the expression of PCNA and serves as a key regulator in bladder cancer development and progression. In conclusion, these findings demonstrated that CCEPR plays an important regulatory role in bladder cancer and may provide a promising diagnostic and therapeutic target in bladder cancer.

## MATERIALS AND METHODS

### Patients and clinical samples collection

55 patients with urothelial carcinoma of bladder who received radical or partial cystectomy were included in this study. After radical or partial cystectomy bladder cancer tissues and normal bladder tissues from each patient were snap-frozen in liquid nitrogen immediately. All patients included in this study signed informed consent and this study was approved by the Institutional Review Board of Peking University First Hospital and Shenzhen University First Hospital.

### Bladder cancer cell lines and cell culture

Bladder cancer cell lines used in this study were purchased from the Institute of Cell Research, Chinese Academy of Sciences, Shanghai, China. The SV-HUC-1 cells were cultured in DMEM Medium (Invitrogen, Carlsbad, CA, USA). The 5637 and SW780 cells were cultured in RPMI-1640 Medium (Invitrogen, Carlsbad, CA, USA).

### siRNA and pcDNA transfection

The specific small interfering RNA and expression vector used in this study were purchased from GenePharma, Shanghai, China. The target sequence of siRNA-CCEPR was 5′- CGAGGGCGAGCATGTTTGTTGTTTA -3′ [31]. The cells were transiently transfected with corresponding siRNA or pcDNA using Lipofectamine 3000 Transfection Reagent (Invitrogen, Carlsbad, CA, USA).

### RNA extraction and quantitative real-time PCR

The total RNA of the tissue samples and cells were extracted using the Trizol reagent (Invitrogen, Carlsbad, CA, USA). The detailed primer sequences included in this study are shown in Supplementary Table 2. Quantitative real-time PCR was performed using the ABI PRISM 7000 Fluorescent Quantitative PCR System according to the manufacturer's instructions.

### Cell counting Kit-8 assay

Cell proliferation was evaluated by Cell Counting Kit-8 (Beyotime Inst Biotech, China) following the Beyotime's instructions. Briefly, 5 × 10^3^ cells per well were seeded in a 96-well plate, then transfected with corresponding siRNA or pcDNA. Finally, the absorbance was finally evaluated at a wavelength of 450 nm using a microplate reader.

### Ethynyl-2-deoxyuridine (EdU) incorporation assay

Cell proliferation was also evaluated by Ethynyl-2-deoxyuridine incorporation assay (Ribo Bio, Guangzhou, China) according to the Ribo Bio's instructions. Briefly, after transfected with corresponding siRNA or pcDNA cells were incubated with 100 μl of 50 μM EdU per well for 2 h at 37°C, respectively. Finally, the cells were visualized under a fluorescence microscopy.

### Cleaved caspase-3 ELISA assay

Cell apoptosis was evaluated by ELISA assay. Briefly, 5 × 10^5^ cells per well were seeded in a 6-well plate, then transfected with corresponding siRNA or pcDNA, respectively. At 48 h after transfection, Cell cleaved caspase-3 activity was measured using the Caspase-3 Colorimetric Assay kit (Abcam, Cambridge, UK) following the Abcam's instructions.

### Flow cytometry analysis

Cell apoptosis and cell cycle were evaluated by Flow cytometry. Cells were collected after transfection for 48 h. Cell apoptosis was determined using PE Annexin V apoptosis detection kits (BD Pharmingen, San Diego, CA, USA) following the instructions. Cell cycle analysis was determined using propidium iodide cell cycle detected kits (BD Pharmingen) according to the instructions. Finally, cell apoptosis and cell cycle were determined using flow cytometry.

### Western blotting analysis

Total protein was separated by sodium dodecyl sulfate-polyacrylamide gel electrophoresis and transferred onto nitrocellulose membranes. Then the membrane was blocked with 5% non-fat milk and incubated with primary antibodies at 4°C overnight. After incubation with specific antibodies (Abcam, Hong Kong, China), the blots were incubated with goat anti-rabbit secondary antibody (Abcam, Hong Kong, China) and visualized with enhanced chemiluminescence.

### Wound healing assay

Cell motility was evaluated by wound healing assay. At 24 h after transfection, a wound field was created using a sterile 200 μl pipette tip. The cells were incubated for 24 h at 37°C, and then the migration of cells were visualized under a digital camera system.

### Transwell assay

The invasion of bladder cancer cells was evaluated by a transwell insert (8 μm, Corning). At 24 h after transfection, 5 × 10^4^ cells were starved in 200 ml serum free medium and placed in the dishes. The lower chamber was filled with 500 ml of complete medium. Then the cells that had migrated to the bottom surface of the filter membrane were stained with 0.5% crystal violet solution and photographed. Finally, the absorbance were evaluated using an microplate reader.

### Statistical analyses

All experimental data from three independent experiments were analyzed by Student's *t*-test or χ2 test with SPSS version 19.0 software (SPSS Inc. Chicago, IL, USA). Results were expressed as mean ± standard deviation and *P*-values of less than 0.05 were considered to be statistically significant.

## SUPPLEMENTARY MATERIALS TABLES


