# Correction: Increased expression of long non-coding RNA CCEPR is associated with poor prognosis and promotes tumorigenesis in urothelial bladder carcinoma

**DOI:** 10.18632/oncotarget.28347

**Published:** 2023-03-21

**Authors:** Yonghao Zhan, Yifan Li, Bao Guan, Xiaoying Chen, Zhicong Chen, Anbang He, Shiming He, Yanqing Gong, Ding Peng, Yuchen Liu, Zhiming Cai, Xuesong Li, Liqun Zhou

**Affiliations:** ^1^Department of Urology, Peking University First Hospital, The Institute of Urology, Peking University, National Urological Cancer Centre, Beijing, 100034, China; ^2^Department of Urology, State Engineering Laboratory of Medical Key Technologies Application of Synthetic Biology, Key Laboratory of Medical Reprogramming Technology, Shenzhen Second People’s Hospital, The First Affiliated Hospital of Shenzhen University, Shenzhen, 518035, China; ^*^These authors have contributed equally to this work

**This article has been corrected:** In Figure 6A, the 4 upper left panel images under the ‘5637’ heading were mistakenly selected from the wrong files. The corrected Figure 6A, obtained using the original data, is shown below. The authors declare that these corrections do not change the results or conclusions of this paper.

Original article: Oncotarget. 2017; 8:44326–44334. 44326-44334. https://doi.org/10.18632/oncotarget.17872

**Figure 6:**
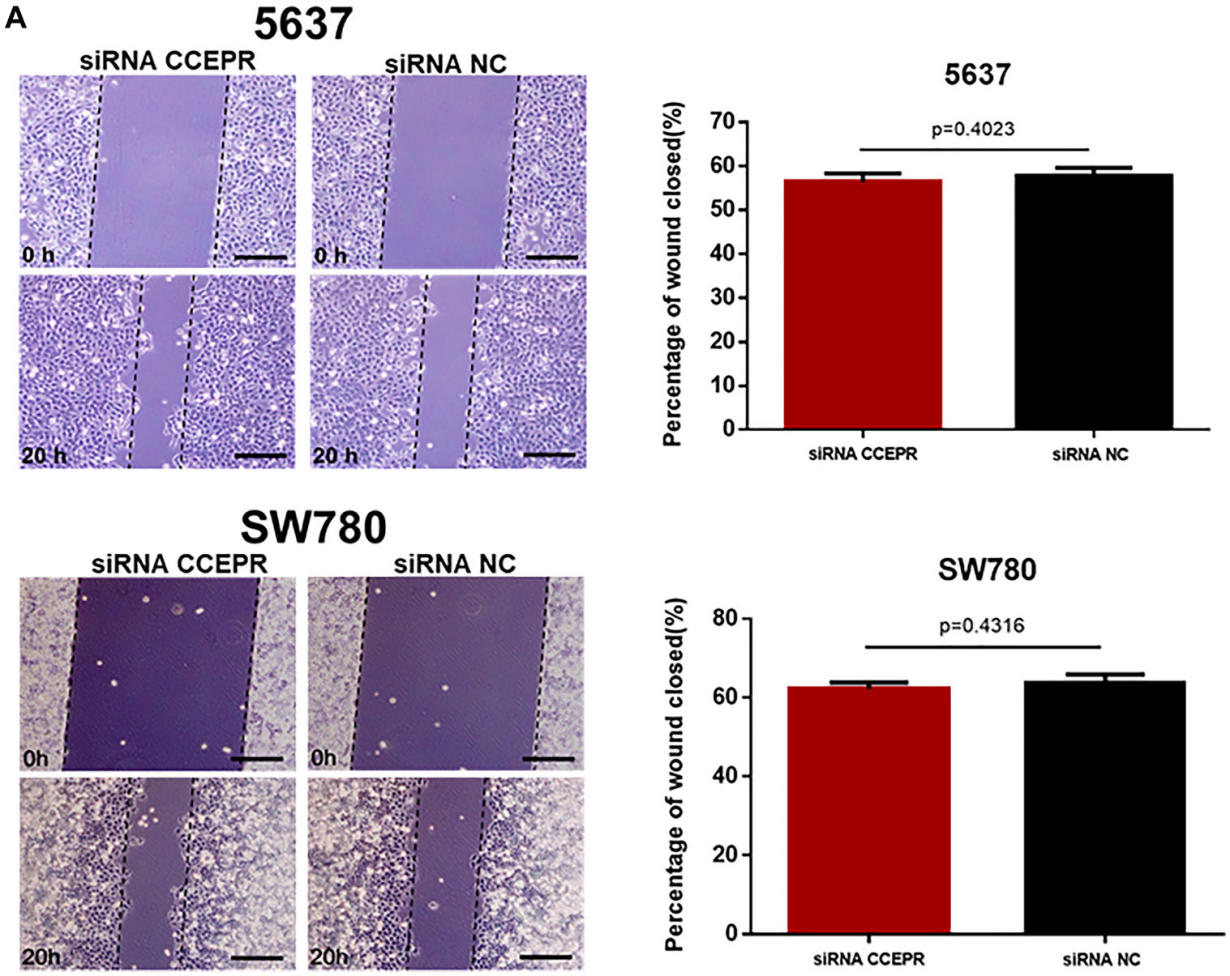
The effect of CCEPR on bladder cancer cell metastasis. (**A** and **B**) There was no significant difference in the migratory ability of bladder cancer cells transfected with corresponding specific siRNA/pcDNA3.1. (**C** and **D**) There was no significant difference in the invasive ability of bladder cancer cells transfected with corresponding specific siRNA/pcDNA3.1. Data are shown as mean ± SD. ^*^ *p* < 0.05; ^**^ *p* < 0.01.

