# Supplementary material for: Increased expression of long non-coding RNA CCEPR is associated with poor prognosis and promotes tumorigenesis in urothelial bladder carcinoma
Source: Oncotarget. 2017 May 15;8(27):44326–34. doi: 10.18632/oncotarget.17872 (PMC5546483; doi:10.18632/oncotarget.17872)
Supplement: Supplementary file 1 [file oncotarget-08-44326-s001.pdf]

# Increased expression of long non-coding RNA CCEPR is associated with poor prognosis and promotes tumorigenesis in urothelial bladder carcinoma

## Supplementary Materials

**Supplementary Table 1: Summary of clinicopathological features of tissues of bladder cancer**

| Pt No. | Sex | Age | Stage | Grade | Pt No. | Sex | Age | Stage | Grade |
|---|---|---|---|---|---|---|---|---|---|
| 1 | M | 66 | T2bN0M0 | H | 29 | M | 58 | T4aN0M0 | H |
| 2 | M | 53 | T1N0M0 | L | 30 | M | 63 | T2aN0M0 | L |
| 3 | M | 75 | T2bN0M0 | H | 31 | M | 50 | T2bN0M0 | H |
| 4 | F | 64 | T1N0M0 | L | 32 | M | 73 | T3bN0M0 | H |
| 5 | M | 58 | T3aN0M0 | H | 33 | F | 62 | T4aN0M0 | H |
| 6 | M | 65 | T2bN0M0 | H | 34 | M | 41 | T1N0M0 | L |
| 7 | F | 38 | T3aN0M0 | H | 35 | M | 62 | T4aN0M0 | H |
| 8 | M | 59 | T2bN0M0 | H | 36 | M | 76 | T2bN0M0 | L |
| 9 | M | 43 | T3aN0M0 | H | 37 | M | 25 | T1N0M0 | L |
| 10 | F | 64 | T2bN0M0 | H | 38 | F | 74 | T3aN0M0 | H |
| 11 | M | 69 | T1N0M0 | L | 39 | F | 70 | T1N0M0 | L |
| 12 | M | 72 | T3aN0M0 | H | 40 | M | 59 | T4N0M0 | H |
| 13 | F | 89 | T1N0M0 | L | 41 | F | 72 | T1N0M0 | L |
| 14 | M | 68 | T2bN0M0 | H | 42 | M | 46 | T1N0M0 | L |
| 15 | F | 63 | T3aN0M0 | H | 43 | M | 63 | T3aN0M0 | H |
| 16 | M | 63 | T2bN0M0 | H | 44 | M | 86 | T1N0M0 | L |
| 17 | M | 78 | T2aN0M0 | L | 45 | M | 70 | T2bN0M0 | H |
| 18 | M | 70 | T2aN0M0 | L | 46 | M | 49 | T1N0M0 | L |
| 19 | F | 41 | T2aN0M0 | L | 47 | M | 61 | T3aN0M0 | H |
| 20 | M | 59 | T2bN0M0 | H | 48 | M | 53 | T2aN0M0 | L |
| 21 | F | 73 | T2aN0M0 | L | 49 | M | 73 | T2bN1M0 | H |
| 22 | M | 67 | T2bN0M0 | H | 50 | M | 47 | T2aN0M0 | L |
| 23 | F | 61 | T3aN0M0 | H | 51 | M | 77 | T3aN0M0 | H |
| 24 | F | 51 | T1N0M0 | L | 52 | M | 66 | T1N0M0 | L |
| 25 | M | 58 | T4aN3M0 | H | 53 | F | 74 | T2bN0M0 | H |
| 26 | M | 63 | T2aN0M0 | L | 54 | F | 60 | T2aN0M0 | H |
| 27 | M | 57 | T4aN0M0 | H | 55 | M | 68 | T1N0M0 | L |
| 28 | M | 54 | T2bN0M0 | H | | | | | |

Pt No. patient number; M male; F female; Grade the World Health Organization 2004 classification; H high; L low; Stage the American Joint Committee on Cancer TNM classification.

**Supplementary Table 2: The primer sequences included in this study**

| Name | primer sequences (5′–3′) |
|---|---|
| CCEPR forward | AAGGTCCCAGGATACTCGC |
| CCEPR reverse | GTGTCGTGGACTGGCAAAAT |
| PCNA forward | GCCATATTGGAGATGCTGT |
| PCNA reverse | TGAGTGTCACCGTTGAAGA |
| GAPDH forward | CGCTCTCTGCTCCTCCTGTTC |
| GAPDH reverse | ATCCGTTGACTCCGACCTTCAC |
